# Mental Representation of Arm Motion Dynamics in Children and Adolescents

**DOI:** 10.1371/journal.pone.0073042

**Published:** 2013-08-29

**Authors:** Lionel Crognier, Xanthi Skoura, Annie Vinter, Charalambos Papaxanthis

**Affiliations:** 1 Université de Bourgogne, Unité de Formation et de Recherche en Sciences et Techniques des Activités Physiques et Sportives, Dijon, France; 2 Institut National de la Santé et de la Recherche Médicale (INSERM), Unité 1093, Cognition, Action, et Plasticité Sensorimotrice, Dijon, France; 3 Université de Bourgogne, Laboratoire d'Etude de l'Apprentissage et du Développement (LEAD), CNRS, UMR 5022, Dijon, France; University of Bologna, Italy

## Abstract

Motor imagery, i.e., a mental state during which an individual internally represents an action without any overt motor output, is a potential tool to investigate action representation during development. Here, we took advantage of the inertial anisotropy phenomenon to investigate whether children can generate accurate motor predictions for movements with varying dynamics. Children (9 and 11 years), adolescents (14 years) and young adults (21 years) carried-out actual and mental arm movements in two different directions in the horizontal plane: rightwards (low inertia) and leftwards (high inertia). We recorded and compared actual and mental movement times. We found that actual movement times were greater for leftward than rightward arm movements in all groups. For mental movements, differences between leftward versus rightward movements were observed in the adults and adolescents, but not among the children. Furthermore, significant differences between actual and mental times were found at 9 and 11 years of age in the leftward direction. The ratio R/L (rightward direction/leftward direction), which indicates temporal differences between low inertia and high inertia movements, was inferior to 1 at all ages, except for the mental movements at 9 years of age, indicating than actual and mental movements were shorter for the rightward than leftward direction. Interestingly, while the ratio R/L of actual movements was constant across ages, it gradually decreased with age for mental movements. The ratio A/M (actual movement/mental movement), which indicates temporal differences between actual and mental movements, was near to 1 in the adults' groups, denoting accurate mental timing. In children and adolescents, an underestimation of mental movement times appeared for the leftward movements only. However, this overestimation gradually decreased with age. Our results showed a refinement in the motor imagery ability during development. Action representation reached maturation at adolescence, during which mental actions were tightly related to their actual production.

## Introduction

Motor imagery is a mental state during which an individual internally represents an action without any overt motor output. This phenomenological experience implies that individuals feel themselves performing a movement in a first-person perspective (e.g., imagined sensation of kicking a ball). Several investigations have provided strong evidence for various neurocognitive similarities between mental and sensorimotor states. For instance, behavioural studies have shown that the required time to produce a specific movement is tightly correlated with the required time to mentally simulate the same movement [1]–[10]. Furthermore, autonomic activation increases proportionally to the mental effort produced during imagined movements [11]–[13] and appropriate mental training enhances motor performance [14]–[19]. Lastly, the activation pattern of brain areas occurring during motor production is broadly shared during the mental simulation state. Notably, the parietal and prefrontal cortices, the supplementary motor area, the premotor and primary motor cortices, the basal ganglia and the cerebellum are activated during both executed and imagined movements [20]–[25].

Motor imagery is a potential tool to investigate action representation [26]. It has been posited that motor prediction is the functional mechanism underling motor imagery process [27]. Motor predictions are generated by internal forward models; that is, neural networks that simulate the dynamic behaviour of the body and its interaction with the environment. For example, during an arm reaching movement, the forward model relates the sensory signals of the actual state of the arm (e.g. position, time, velocity) to the copy of motor commands (efferent copy) and predicts the future states of the arm (forward dynamic model) and the sensory consequences of its motion (forward sensory model). This prediction can thus be used to monitor whether an ongoing movement proceeds as planned. Childhood is a decisive period for the development of internal models. The brain area that monitors intentions and plans at high levels of representation is thought to be the parietal cortex [8], [22], [24], [28], [29]. Neuroimaging findings have shown that the parietal cortex undergoes a particularly extended course of development, compared with sensory and motor regions of the brain [30]–[32], which may explain the increasing ability with age to mentally predict future sensorimotor states. At the psychophysical level, recent studies have addressed the chronometry of executed and imagined hand actions in children and adolescents using several motor imagery tasks. A key finding is that the ability to mentally evoke a motor image emerges around 7-years-olds when children are able to think about themselves [33]. Moreover, correlations between imagined and executed movements are low in the very young participants, but gradually increase across age until adolescence [34]–[39]. Precisely, the normal speed-accuracy trade-off (Fitts' Law task), which posits that we slow down when we wish to increase accuracy of movements, is acquired at adolescence after a gradual improvement in childhood. These age-related changes in motor imagery ability may reflect the children's emerging ability to represent internal models for prospective actions.

All the previous studies have investigated the development of internal models through imagined actions by predominantly manipulating kinematic variables, such as the speed-accuracy trade-off. One interesting question, however, is whether the gradual improvement in motor imagery ability from childhood-to-adolescence also holds in motor tasks with dynamic constraints. How does the brain build actions representation of body dynamics during development? Is the temporal correlation between executed and imagined actions during development similar for tasks with spatiotemporal and dynamic constraints?

**Figure 1:**
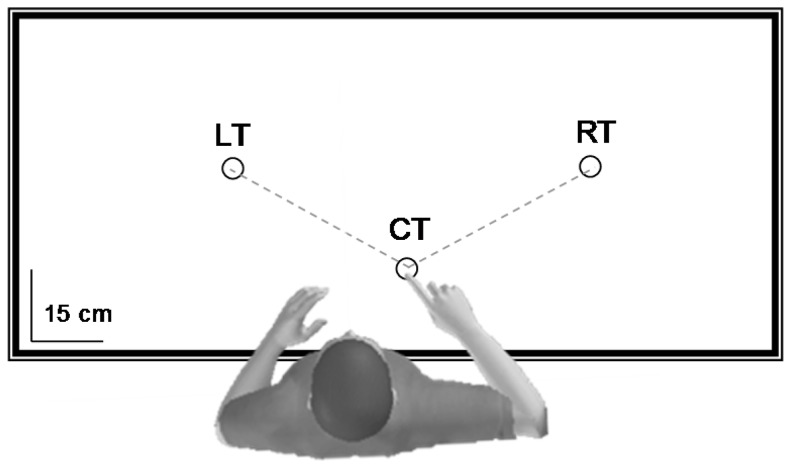
Experimental device (top view). Children, adolescent and young adults performed horizontal arm pointing movements toward two targets placed on the right (60°, RT) and on the left (60°, LT) of the central target (CT). The target CT indicated the starting position. Inertial resistance was low in the direction CT-RT and high in the direction CT-LT.

**Figure 2:**
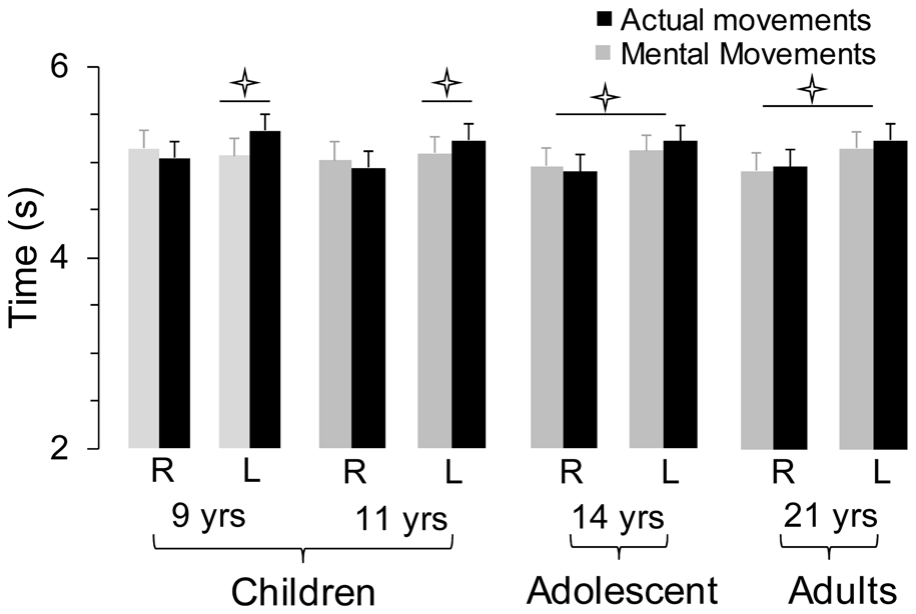
Average values (+SE) of actual and imagined movement times are illustrated for the four age-groups and the two directions (L, left and R, right). Diamonds indicate significant differences between actual and mental movements. Stars indicate significant differences between right and left directions for both actual and mental movements.

In the current study, we took advantage of the inertial anisotropy phenomenon to investigate whether children can generate accurate motor predictions for movements with varying dynamics. In a two-joint mechanical system such as the upper-arm and the forearm, motion dynamics change according to movement direction [4], [40], [41]. For instance, when we reach with our right arm rightwards the movement is accomplished principally by the motion of the forearm; consequently, the mass of the upper arm contributes little to the total inertia of the arm. On the contrary, when we perform the same movement leftwards, we move both the upper-arm and forearm, increasing consequently the total inertia of the arm (see Fig. 1 and 2 for detailed explanations). Previous studies have reported that direction-dependent changes in arm dynamics influence movement time [4], [40], [42]. Specifically, for the same hand amplitudes, arm movements with high inertia are slower than arm movements with low inertia. It is important to note that the brain maintains accurate internal representations of the inertial anisotropy of the arm. For example, by means of a grip-force/load-force coupling paradigm, it has have been demonstrated that the brain accurately anticipates the inertial anisotropy of the right arm and therefore the direction-dependent changes in movement time [43]. Similarly, it has been shown that young adults accurately integrate these direction-depended temporal asymmetries into the motor imagery process [4], [42]. Specifically, actual and mental movement times were equivalent whatever the direction of the movement and both actual and mental movement times were shorter for lower inertia than high inertia directions.

In the present experiment, children, adolescents and adults performed actual and mental arm movements in two different directions with varying dynamics. We recorded actual and mental movement times and fixed two criteria for arguing in favour of accurate representations of body dynamics in children: first, actual and mental movement times should be equivalent for both directions (low and high inertia); second, actual and mental times of rightward movements (low inertia) should be shorter than actual and mental times of leftward movements (high inertia). Regarding previous studies that demonstrated a development of the parietal cortex until adolescence [30], [31], [44], [45] and considering that this brain region is important for the generation of motor predictions [8], [28], we anticipated that action representation would exhibit progressive improvement with age until adolescence, at which mental and sensorimotor states will overlap.

## Methods

### Ethical statement

All adult participants gave their written informed consent prior to their inclusion in this study. Written parental consent was obtained for each child and adolescent. The experimental protocol was approved by the ethics committee of Burgundy and was carried out in agreement with legal requirements and international norms (Declaration of Helsinki, 1964).

### Participants

Two children groups, the 9-year-olds (9 female and 9 male; mean age: 8.9±0.3 years) and the 11-year-olds (9 female and 9 male; 10.8±0.4 years), one group of adolescents (10 female and 8 male; 13.7±0.3 years), and one group of adults (10 female and 8 male; mean age: 21.2±0.4 years) participated in the present experiment. Children and adolescent were essentially from middle socioeconomic status families, and their parents reported that all were in good health, with normal or corrected vision and without any neuromuscular, cognitive and learning disorders. The young group was composed from students from the University of Burgundy who reported being in good health with normal or corrected vision and without any neuromuscular disorders.

### Assessment of Imagery ability and Arm Preference

Adults' general motor imagery ability was assessed by means of a French version of the Movement Imagery Questionnaire [46]. All adults were good imagers as they reported imagery scores higher than 40 (maximum score 56). General motor imagery ability of children and adolescent was evaluated by using an adapted version (12 questions) of the Florida praxis Imagery Questionnaire (FPIQ) [47]. There were no differences between the two children groups and the adolescent group (independent samples t-tests, t<1, p>0.1) in their ability of imaging several actions (9 yrs: 86% correct responses; 11 yrs: 89% correct responses; 14 yrs: 94% correct responses).

Right arm preference in children was assessed by means of simple tests drawn from the handedness protocol described by Bryden [48] **.** Eight items were used, 4 were unimanual (drawing, throwing a ball, holding scissors and brushing one's teeth) and 4 were bimanual (tightening the lid on a bottle, hitting a nail with a hammer, lighting a match and wiping a plate with a cloth). Only children who obtained a score equal or superior to 6 were selected (6 children were eliminated from the sixty initially included in the study). Right arm preference in young adults (mean laterality 0.85±0.04) was determined by means of the Edinburgh Handedness inventory [49].

### Material and Experimental procedure

Participants were comfortably seated on an adjustable chair in front of a table whose edge was aligned with their chest at the level of the diaphragm. Three targets (black circles, 2 cm diameter) were drawn on the table (see Fig. 1). The central target (CT) indicated the starting position and was placed 20 cm forward from the participants' right shoulder. The two other targets were placed at the right (RT, 60°) and at the left (LT, 60°) of the CT at a distance corresponding to 90% of the total length of their right arm. The angle between the three targets was 120°. The participants, using their right arm, were requested to actually move or mentally simulate moving (imagined movement) at a natural self-selected speed between the CT and RT or between the CT and LT. Actual and mental trials were performed with eyes open. It is known that relatively long trial durations are necessary to obtain reliable measurements in motor imagery protocols because movement durations have a coarse resolution [16], [50]. Therefore, in our protocol one trial corresponded to three successive and fluid arm movements between the CT and the other targets: CT-RT-CT-RT-CT-RT-CT and CT-LT-CT-LT-CT-LT-CT. For the actual trials, participants were asked to move their arm over the table without touching it. For the mental trials, participants were requested to place their arm above the CT, to keep it motionless during the whole trial and to feel themselves performing the task from a first-person perspective, as they would actually do [34]. Imagining a movement in the first person is a necessary condition to engage the motor system [51].

Prior to the experiment and after receiving a demonstration by the experimenter, all participants actually practiced 3 times. The results of these trials were not included in the main experiment. All participants reported having understood the task requirements and none of them had expressed any inconvenience regarding its actual performance. Mental practice trials were also performed. After having practiced 6 to 10 times, all participants reported being able to imagine the movement in a first-person perspective. During the experiment, each participant performed 12 actual and 12 mental trials in each direction; all trials were randomized. No information was given to participants concerning their temporal performance. A rest of 1 min was introduced after 10 trials. The experiment lasted ∼30 minutes per participant and none of them reported any muscular or mental fatigue.

### Data recording and Statistical analysis

The time of actual and mental movements was recorded by means of an electronic stopwatch (temporal resolution 1 ms). The time interval between the experimenter's go signal and the participant's stop signal was measured. This method gave consistent results in previous experiments [34]. For each participant, the mean time and its standard error (SE) were calculated for each experimental condition separately. Variables were normally distributed (Shapiro-Wilk W test, P>0.05) and their variance was equivalent (Levene's test, P>0.05). We performed ANOVA with *age* as a between-subjects factor (9 yrs, 11 yrs, 14 yrs, Adults), and *direction* (R, L) and *movement* (mental, actual) as within-subjects factors. *Post hoc* differences were assessed by means of Newman-Keuls tests. Statistical effects were considered as significant at p<0.05.

To evaluate temporal differences between low inertia (LI) and high inertia (HI) movements, we calculated, for each participant, the ratio (HI/LI) of the average time of high inertia movements (n = 12 trials) and the average time of low inertia movements (n = 12 trials). A ratio near to one (1) indicates equivalent times for high inertia and low inertia movements. Similarly, to examine temporal differences between actual (A) and mental (M) movements, we computed the ratio (A/M) of the average time of actual movements (n = 12 trials) and the average time of mental (n = 12 trials) movements. A ratio near to one (1) indicated equivalent times for actual and mental movements. We checked that these variables were normally distributed and that their variance was equivalent. Statistical differences for the HI/LI and A/M were tested using ANOVAs with age as a between-subjects factor and direction as within-subject factors. *Post hoc* differences were assessed by means of Newman-Keuls tests, and size effects (*n^2^_p_ value)* were assessed for each significant effect.

## Results

Average times (+SE) of actual and mental movements are illustrated in Fig. 2 for the four age groups and the two movement directions. The ANOVA revealed a main effect of *direction* (F_1,68_ = 128.24, *n^2^_p_* = .65, p<0.0001); times were greater for leftward (on average 5.20±0.17 s) than rightward (on average 4.95±0.16 s) movements. There was also an interaction effect between *direction* and *movement* (F_1,68_ = 35.98, *n^2^_p_* = .34, p<0.0001); actual and mental times differed for leftward (p<0.0001) but not for rightward (p = 0.20) movements. The interaction between *age* and *direction* was also significant (F_3,68_ = 4.42, *n^2^_p_* = .16, p<0.001); leftwards and rightwards movements significantly differed (p<0.001) for the age of 9 years only. There was also a significant interaction effect between *age*, *direction* and *movement* (F_3,68_ = 4.42, *n^2^_p_* = .16, p = 0.007). *Post hoc* analyses showed that actual times were greater for leftward (high inertia) and rightward (low inertia) arm movements in all groups (in all cases, p<0.001). For mental movements, differences between leftward versus rightward movements were observed in the adults' (p<0.001) and 14 yr-olds' (p = 0.01) groups, but not in the 11 (p = 0.38) and 9 yr-olds' (p = 0.37) groups. Furthermore, significant differences between actual and mental times were found at 9 (p = 0.01) and 11 years of age (p = 0.04) in the leftward direction. Table 1 shows main and interactions effects of the ANOVA analysis.

**Table 1:** Main and interaction effects of the ANOVA analysis.

Statistical Effects	Factors	Statistical Results
Main effects	Age	F_3,68_ = 0.07, p = 0.97
	Direction *	F_1,68_ = 128.24, p<0.0001
	Movement	F_1,68_ = 1.51, p = 0.21
Two-way interactions	Age × Movement	F_3,68_ = 0.12, p = 0.94
	Direction × Movement *	F_1,68_ = 35.98, p<0.0001
	Age × Direction *	F_3,68_ = 4.42, p<0.001
Three-way interactions	Age × Movement × Direction *	F_3,68_ = 4.42, p = 0.007

* Indicate significant effects.

**Figure 3:**
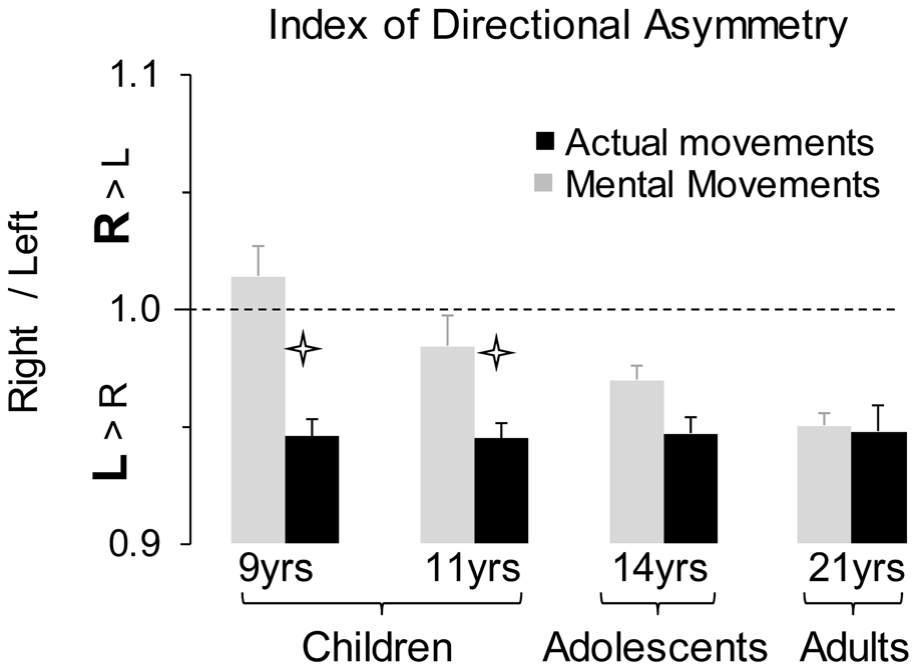
Average values (+SE) of the index of directional asymmetry (R/L) for the four age-groups. Stars indicate significant differences between actual and mental movements.

Fig. 3 shows average values (+SE) of the ratio R/L. It is noticeable that the values were inferior to 1, except for the mental movements at 9 years of age, indicating than actual and mental movements were shorter for the rightward (low inertia) than leftward (low inertia) direction. Interestingly, while the ratio R/L of actual movements was constant across ages (see black histograms in Fig. 3), it gradually decreased with age for mental movements (see grey histograms in Fig. 3). The ANOVA confirmed these observations by revealing a significant interaction between *age* and *movement* (F_3,68_ = 12.36, *n^2^_p_* = .35, p<0.0001). *Post hoc* analysis showed that the ratio R/L in mental movements significantly decreased with age (for all comparisons p<0.05; but 14 yrs versus adults, p = 0.84), while it was constant in actual movements (in all cases, p>0.5). Furthermore, the ratio R/L differed between actual and mental movements at 9 (p<0.001) and 11 years of age (p = 0.03), but not at 14 years of age (p = 0.10) and in adults (p = 0.83).

**Figure 4:**
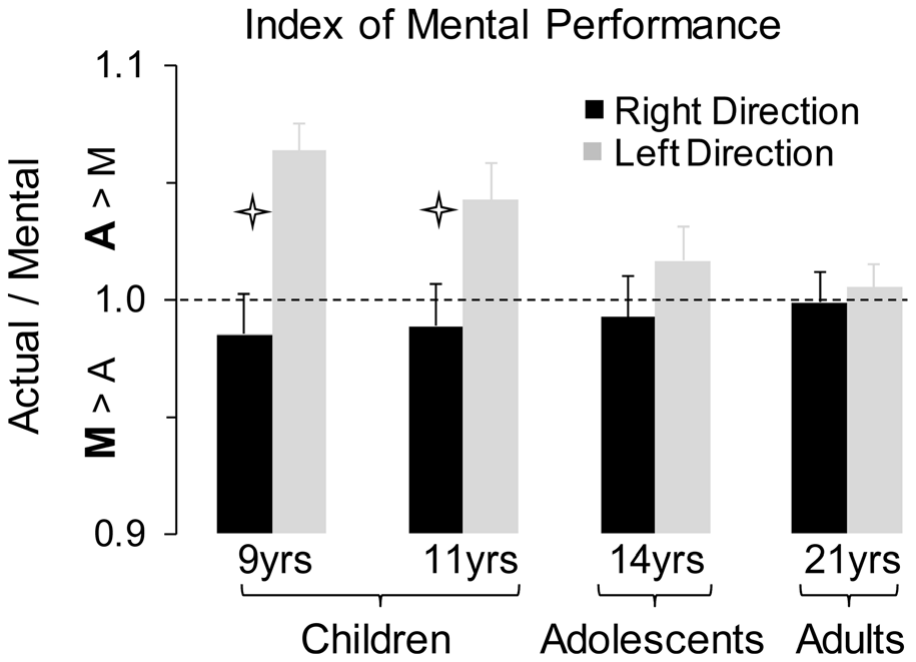
Average values (+SE) of the index of mental performance (M/P) for the four age-groups. Stars indicate significant differences between right and left directions.

Fig. 4 shows the average values (+SE) of the ratio A/M. In adults, these values were near to 1, denoting accurate mental representations for both right (low inertia) and left (high inertia) directions. In children, an overestimation of actual movements appeared for the leftward movements. However, this overestimation gradually decreased with age. The ANOVA revealed an interaction effect between *age* and *direction* (F_3,68_ = 10.84, *n^2^_p_* = .32, p<0.0001). *Post hoc* analysis showed significant differences between rightward and leftward movements at 9 (p = 0.001) and 11 years of age (p = 0.01), but not in the 14-year-olds (p = 0.17) and in adults (p = 0.71). Furthermore, the ratio A/M in leftward direction significantly decreased with age (for all comparisons, p<0.05; except for 9 yrs versus 11 yrs and 14 yrs versus adults, for both p>0.3).

## Discussion

The aim of this study was to investigate the progress of the integration of task dynamics into action representation in children (9 and 11 years), adolescents (14 years), and adults (21 years). The age-dependent improvement of motor imagery process was evaluated by means of the mental chronometry paradigm in a motor task involving direction-dependent inertial constraints (i.e., inertial anisotropy phenomenon). A gradual increase with age in the timing correspondence between actual and mental movements confirmed the refinement in the motor imagery ability during development. Action representation reached maturation at adolescence, during which mental actions were tightly related to their actual production. These findings are interpreted with the theoretical basis of the simulation theory and the concept of internal models.

### Actual movements under dynamic constraints across ages

Arm movements with low inertia (rightward movements) were faster than those with high inertia (leftward movements) in all groups. The index of directional asymmetry (R/L) for actual movements, which indicated the effect of the inertial constraints on movement time, remained constant across ages. Such direction-dependent temporal differences have been reported in earlier investigations and were attributed to the inertial anisotropy phenomenon [4], [40], [42], [43]. The novelty here is that timing variations with respect to movement direction, and therefore with respect to movement dynamics, were observed early during development, before 9 years of age in the present study. It would be interesting in the future to examine at which age kinematic variations with respect to inertial resistance emerge and to compare this evolution with the development of other aspects of movement production, such as joint coordination [52], [53], limb selection [54], and anticipatory postural adjustment [55]. Another interesting finding is that actual movement times were similar for all groups. This suggests that from children to adults task dynamics were well-integrated at the level of movement production.

### Mental movements under dynamic constraints across ages

We found that the temporal features of mental movements emulated those of actual movements in adolescents and adults. Precisely, mental durations were shorter for low inertia (rightwards) than high inertia (leftwards) directions; the index of directional asymmetry (R/L) was inferior to one in both groups. Interestingly, mental and actual arm movement times were similar in both directions. This was further confirmed by the finding that the index of mental performance (A/M) was near to one, suggesting an isochrony between actual and mental movement times. These results indicate that mental representation of action dynamics reached maturation in adolescence, as has been proposed by previous studies [36], [37]. The CNS forms accurate representations of the inertial anisotropy of the arm and makes use of this representation to accurately simulate arm movements [4], [6], [42].

Conversely, temporal features of mental movements in children did not mimic those of actual movements. Specifically, mental times were almost identical for both low inertia (rightwards) and high inertia (leftwards) directions; as if children anticipated similar inertial resistance in the two directions (the ratio R/L was near to one). Interestingly, mental and actual arm movement times were similar for the low inertia direction, but dissimilar for the high inertia direction (see A/M ratio). This novel finding indicates that the integration of motion dynamics into action representation was progressively developed until adolescence. In children, the CNS did not maintain an accurate representation of the inertial anisotropy of the arm and therefore mental movements differed from their actual counterparts. A similar conclusion has been formulated for spatiotemporal constraints. It has been shown that the normal speed-accuracy trade-off (Fitts' Law task), which posits that we slow down when we wish to increase accuracy of movements, is acquired at adolescence after a gradually improvement in childhood [33]–[39]. Our findings and those of previous studies suggest a progressive maturation in action representation with age. Here, it is interesting to mention the discrepancy between the general imagery ability measured by the questionnaire (no difference between children and adolescents were found) and the specific imagery ability required by our task, in which significant differences between groups were detected. This finding may indicate that motor imagery ability in children is task-dependent and general conclusions regarding action representation during development should be carefully drawn.

### Internal models for action and brain development

We suggest that improvement in action representation in childhood may be due to refinement of internal models. Action representation is generated by an internal forward model, which is a neural network that simulates the dynamic behavior of the body and its interaction with the environment [16], [27], [50], [56]. Theoretically, when participants imagine arm movements, the forward model relates the actual state of the arm (e.g. position, time, velocity) to the neural commands (efference copy) prepared by the motor controller and predicts the future states of the arm (forward dynamics model) and their sensory consequences (forward sensory model). When the CNS has an accurate internal representation of limb and environmental dynamics, movement prediction is very close to movement production (in mental actions, this is attested by the well-known isochrony) and, theoretically, movement can be controlled in feed-forward without requiring on-line feedback regulation [1], [4], [7], [57], [58]. If internal representations of limb and/or environmental dynamics are biased or variable, a discrepancy between state estimation and actual state could emerge [50], [59]–[61]. Here, we found a discrepancy between imagined movement time (estimated state) and actual movement time (actual state) in children. This suggests that children have not completely acquired this ability, as they exhibited temporal differences between executed and imagined arm movements. However, our findings clearly showed that motor imagery capacity progressively improved with age. This indicates that internal forward models became more reliable with experience and practice. Since during development internal models for action are not yet completely acquired, sensory feedback must play an important role for the control of action.

The parietal cortex and the cerebellum, although their differential roles are not clear, have been proposed to play a role in sensorimotor prediction [28]. In particular, the parietal cortex plays a major role in mental rehearsal of motor actions [8], [28], [29], [36], [37], [62]–[64]. For instance, impairment in imagined actions occurs in patients with lesions in the parietal cortex [8]. One could speculate that inaccuracy in action representation in children is related to maturational processes, i.e. grey and white matter development in the parietal cortex [30], [31], [44], [45]. With advance in age, the maturational processes in parietal cortex increases neural efficiency and may help oldest children and adolescents to progressively improve their ability to generate accurate motor predictions [32], [34], [36], [37].

### Limitations and perspectives

Findings from this study are consistent with those from other investigations suggesting the refinement of internal models during the transition from childhood to adolescence [34]–[37]. However, testing children introduces some methodological factors that may affect the interpretation of our results. For instance, during motor imagery children need to maintain a high level of concentration. Although specific instructions and appropriate training (e.g., training session to build a motor image of the required movement) were given to children, we cannot rule out that some cognitive factors, such as attention or concentration, may have at least indirect effects on children's performance in the imagined motor task. Another interesting point, which needs further exploration, is related to inter-individuals differences at the motor level. Although not tested here, the regular practice of a physical activity, such as playing a sport or a musical instrument, may substantially contribute to the improvement of motor imagery ability.
